# *I*mproving *m*edication *p*rescribing-*r*elated *o*utcomes for *v*ulnerable *e*lderly *i*n *t*ransitions on *h*igh-*r*isk *m*edications (*IMPROVE-IT HRM*): a pilot randomized trial protocol

**DOI:** 10.1186/s40814-024-01484-6

**Published:** 2024-04-10

**Authors:** Anne Holbrook, Dan Perri, Mitch Levine, Lawrence Mbuagbaw, Sarah Jarmain, Lehana Thabane, Jean-Eric Tarride, Lisa Dolovich, Sylvia Hyland, Victoria Telford, Jessyca Silva, Carmine Nieuwstraten

**Affiliations:** 1 Division of Clinical Pharmacology and Toxicology, Department of Medicine, McMaster University, Hamilton, ON Canada; 2 Clinical Pharmacology Research, Research Institute of St. Joes Hamilton, Hamilton, ON Canada; 3 Department of Health Research Methods, Evidence and Impact (HEI), McMaster University, Hamilton, ON Canada; 4 Digital Solutions, St. Joseph’s Healthcare Hamilton, Hamilton, Canada; 5 Department of Anesthesia, McMaster University, Hamilton, ON Canada; 6 Department of Pediatrics, McMaster University, Hamilton, ON Canada; 7 Biotatistics Unit, Research Institute of St. Joes Hamilton, Hamilton, ON Canada; 8 Centre for Development of Best Practices in Health (CDBPH), Yaoundé Central Hospital, Yaoundé, Cameroon; 9 Division of Epidemiology and Biostatistics, Department of Global Health, Stellenbosch University, Cape Town, South Africa; 10 Medical and Academic Affairs, St. Joseph’s Healthcare Hamilton, Hamilton, ON Canada; 11 Faculty of Health Sciences, University of Johannesburg, Johannesburg, South Africa; 12 Center for Health Economic and Policy Analysis, McMaster University, Hamilton, ON Canada; 13 Programs for Assessment of Technology in Health (PATH), The Research Institute of St. Joes Hamilton, Hamilton, ON Canada; 14 Leslie Dan Faculty of Pharmacy, University of Toronto, Toronto, ON Canada; 15 Department of Family Medicine, McMaster University, Hamilton, ON Canada; 16 Institute for Safe Medication Practices Canada, North York, ON Canada; 17 Pharmacy Services, St. Joseph’s Healthcare Hamilton, Hamilton, ON Canada

**Keywords:** Medication management, Hospital discharge, High-cost user, Seniors, Clinical pharmacology, Pilot RCT, Medication safety, Major polypharmacy, High-risk medications

## Abstract

**Background:**

Seniors with recurrent hospitalizations who are taking multiple medications including high-risk medications are at particular risk for serious adverse medication events. We will assess whether an expert Clinical Pharmacology and Toxicology (CPT) medication management intervention during hospitalization with follow-up post-discharge and communication with circle of care is feasible and can decrease drug therapy problems amongst this group.

**Methods:**

The design is a pragmatic pilot randomized trial with 1:1 patient-level concealed randomization with blinded outcome assessment and data analysis. Participants will be adults 65 years and older admitted to internal medicine services for more than 2 days, who have had at least one other hospitalization in the prior year, taking five or more chronic medications including at least one high-risk medication. The CPT intervention identifies medication targets; completes consult, including priorities for improving prescribing negotiated with the patient; starts the care plan; ensures a detailed discharge medication reconciliation and circle-of-care communication; and sees the patient at least twice after hospital discharge via virtual visits to consolidate the care plan in the community. Control group receives usual care. Primary outcomes are feasibility — recruitment, retention, costs, and clinical — number of drug therapy problems improved, with secondary outcomes examining coordination of transitions in care, quality of life, and healthcare utilization and costs. Follow-up is to 3-month posthospital discharge.

**Discussion:**

If results support feasibility of ramp-up and promising clinical outcomes, a follow-up definitive trial will be organized using a developing national platform and medication appropriateness network. Since the intervention allows a very scarce medical specialty expertise to be offered via virtual care, there is potential to improve the safety, outcomes, and cost of care widely.

**Trial registration number:**

ClinicalTrials.gov identifier: NCT04077281.

**Supplementary Information:**

The online version contains supplementary material available at 10.1186/s40814-024-01484-6.

## Background

Current systematic reviews of randomized trials to manage polypharmacy or to manage medications in hospital or in the transitions of care have not consistently shown improvements in important clinical outcomes [1–5]. This is largely because (a) the interventions have been carried out by providers without the requisite combination of diagnostic, therapeutic, and risk management expertise or authority to make requisite changes, (b) the intervention was not sufficiently concentrated (too short, incomplete, or misdirected), (c) the medication focus was misplaced (i.e., not high-risk medications, which are the most associated with adverse clinical outcomes), (d) the outcomes were overly focused on poor quality surrogates for clinical outcomes, or (e) the patient has so much irreversible comorbidity that changes in medications could not have significant impact.

Because of the huge potential burden of harm and the number of affected vulnerable older adults in most countries, the value of more randomized trial inquiry is high. There is enough suggestion of benefit from trials on prescribing appropriateness combined with the potential for major medication safety improvements, to support an expert intervention concentrated on the highest risk group as they transition through a very high-risk period targeting the highest risk medications [4, 6–9]. In addition, recent advances in digital health make it reasonable to focus on utilizing clinical pharmacologist expertise for this high priority problem within robust communication systems that support patient consultation and follow-up from any geography no matter how remote. Clinical pharmacologists are medical specialists, in this case with internal medicine cross-specialization, who regularly lead the care of hospitalized medically complex patients and formulate their therapeutic priorities.

Transitions in care, particularly in- and out-of-hospital and high alert medications, are two primary high-risk medication safety situations referenced by WHO in their Global Patient Safety Challenge [10]. Even with evidence of under-detection, systematic reviews of the literature conclude that adverse drug events (ADEs) amongst older adults lead to approximately 1 in 10 hospitalizations [11–13]. Adverse drug-related hospital admissions appear to be directly correlated with the number of medications taken concurrently, the number of prescribers involved, possibly the number of pharmacies used, and with hospitalization within the previous year [14]. In several studies, female sex was also a risk factor. Once in hospital, patients (*n* = 46,626) remained at high risk of ADEs, with a mean prevalence of 21.6% (*SD* 16.7), 20.7% of these judged to be severe or life threatening and 32.3% (*SD*22.6%) judged to be preventable [15]. In addition to the risk factors above, a complex patient (several comorbidities with several provider experts involved) is significantly more likely to suffer adverse events in hospital [16, 17]. The period immediately following hospital discharge remains high risk with 37% of seniors sustaining medication-related harm (81% serious) within 8 weeks [18]. In this cohort study from the UK, female sex was associated with medication-related harm [18]. The outcomes of ADEs included prolonged length of stay, frequent readmission, emotional trauma, high costs, and death [16, 19, 20].

The prevalence of major polypharmacy defined as the concurrent, regular use of 10 or more medications is high amongst seniors reaching 38.4% in those aged 85 years and older [4, 21]. While the number of medications dispensed remains a useful signal, it is impossible to gauge the quality of medication regimens simply by the number of medications, as this does not account for the patient’s main diagnosis, comorbidities, risk factors, past history of medication use, or the benefit-harm ratio of the drug for that patient. For example, an older patient with diabetes frequently requires two glucose lowering medications, a statin for cholesterol, and two to three medications for blood pressure, just to manage their high cardiovascular risk without treating their other health problems. Thus, the medication safety target is problematic polypharmacy as opposed to appropriate polypharmacy [22].

Medications which frequently lead to harm outweighing benefit in certain situations are termed “potentially inappropriate medications” (PIMs). PIMs that have been associated with ADEs have been grouped together in medication screening lists, with the most evidence-based being the STOPP criteria [23]. Randomized trials in Europe show that use of the STOPP criteria as a trigger for medication review for hospitalized seniors can improve the appropriateness of prescribing, reduce ADEs, and reduce length of stay [24, 25]. A Canadian study found that nearly 40% of seniors fill a prescription for at least one PIM per year, with the highest rates in women > 85 years of age and the most common PIM drug category being sedative-hypnotic drugs [26]. The cost of these PIMS plus the cost of treating their adverse effects was estimated to be more than US $1.8 billion annually [26].

### Priority medications

Although STOPP is an excellent screening tool, there are too many alerts (80 in current iteration) to feasibly apply in hospitalized patients where timely discharge is a high priority [25]. Analyses of Canadian and US data on drug-related causes of hospitalization by our group and others suggest recurring groups of very commonly used medications as the main causes of drug-related hospitalizations [14, 27, 28]. These are mostly medication families with proven benefit of varying clinical importance, but all cause clinically important harm when not managed well. Thus, these are medications that should trigger review of the entire medication regimen to consider improvements. We have labelled these the high-risk medications (HRM). A detailed list is shown in Appendix 1, but the main families are as follows:

i. Anticoagulants
ii. Analgesics including opioids, NSAIDs, and colchicine
iii. Antimicrobials — long term or restricted
iv. Antineoplastic agents
v. Glucose-lowering drugs
vi. Cardiac drugs including diuretics and digoxin
vii. Sedative-hypnotics including benzodiazepine receptor antagonists, trazodone, and baclofen
viii. Antipsychotic agents
ix. Other psychoactive medications including lithium, trazodone, and tricyclic antidepressants
x. Dementia medications
xi. Immune-modulating agents such as corticosteroids

In many cases, the high-risk medication is required for the patient, but the dose may require adjustment, a tapering regimen may reduce the potential for harm, or a review for potentially serious drug interactions or medication burden identifies another medication that can be removed to decrease the patient’s overall risk of medication-related harm. We have previously developed an “Appropriateness of Prescribing Evaluation Questionnaire” that has been validated as a comprehensive medication appropriateness assessment tool [29, 30]. Optimization of high-risk medications, of course, opens up other opportunities including removal of medications and supplements with no benefit or with possibility of contamination or substitution of more cost-effective alternatives.

### Priority patients

High-cost healthcare users have been an international priority target for quality and cost of care improvements for years [31]. We have shown that 5% of 12 million citizens generate 65% of the entire healthcare costs, and approximately 70% of these are seniors with multiple hospital admissions and problematic polypharmacy [32–34]. Use of high-risk medications is very prevalent in this population and strongly predictive of future healthcare utilization and mortality in a dose- and duration-dependent manner compared to nonusers [33–36].

### Priority situations

Senior high-cost users taking high-risk medications who are transitioning into and out of hospital are at very high risk of serious ADEs. Hospitalization is a double-edged sword in that it defines high-risk situations but also houses the expertise required to effectively intervene. This opportunity to optimize medication regimens for these inpatients is widely underutilized worldwide, due to (a) huge pressures to just deal with the main problem requiring admission and get the patient discharged as quickly as possible due to bed shortages and (b) lack of expertise amongst general medicine and surgery admitting services to complete an expert medication assessment quickly. In preparation for this trial, we recently completed a chart review of 100 randomly selected senior high-cost users who were admitted to a Hamilton Hospital (mean age 82 years) and found the mean rate of potentially inappropriate medications to be 2.8 per person [37]. Only 16.6% of these had been addressed by the time of discharge [37].

## Methods

### Detailed research question

Is it feasible for an expert clinical pharmacology team to coordinate and improve medication management during the high-risk transition period from hospitalization through early post-hospital discharge follow-up for seniors who are high-cost healthcare users and taking high-risk medications, to warrant a large subsequent trial?

### Design

**Fig. 1:**
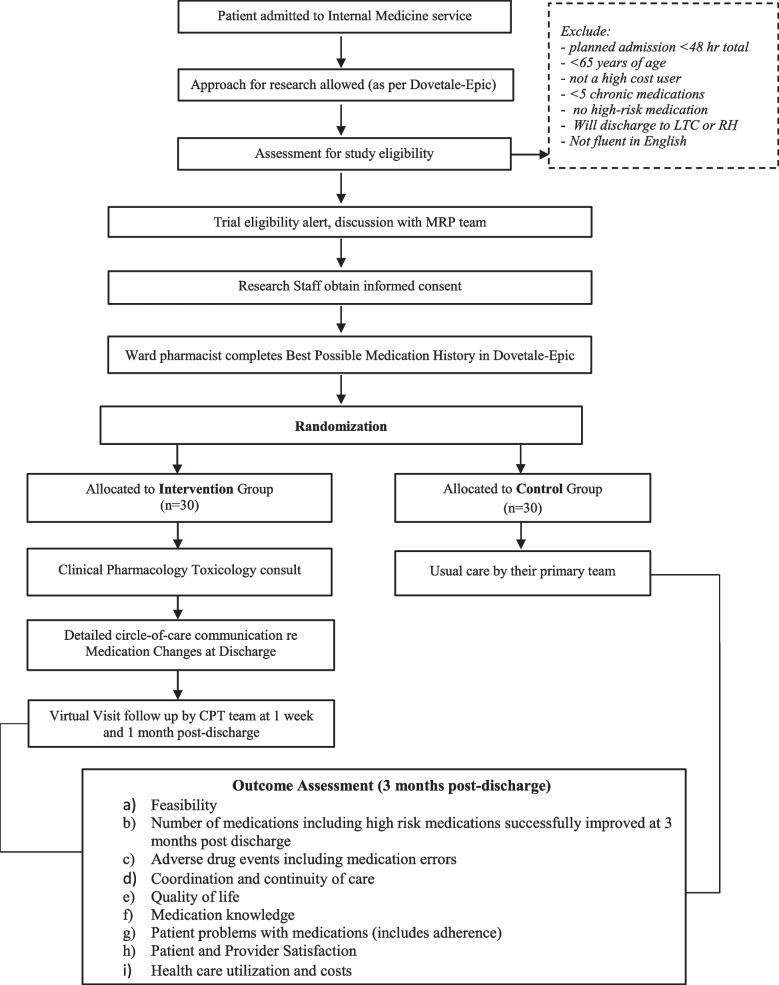
IMPROVE-IT HRM flow diagram

This is a single-center pragmatic pilot randomized trial (RCT) with 1:1 patient-level concealed-allocation randomization with blinded outcome assessment and data analysis [38]. Randomization provides the highest quality methods to minimize bias, the pragmatic design ensures relevance to clinical practice essential for implementation, and a pilot RCT addresses feasibility of a large definitive subsequent RCT directly without waste of research dollars [39, 40]. A study flow diagram is shown below in Fig. 1. Study setting is a 700-bed academic hospital with a busy urgent care and emergency department providing medical, surgical, psychiatric, and obstetric-gynecology inpatient and outpatient care in Hamilton, Ontario.

### Participants

Adults 65 years and older who are admitted to an internal medicine acute care ward with an expected length of stay of more than 2 days, who are high-cost users defined as at least one other hospitalization within the previous year, who are taking five or more chronic medications including at least one high risk medication, and provide informed consent. Patients will be excluded if they are being discharged to long-term care or other setting where they or their caregiver is not in charge of their medications. It is estimated that at least *10 patients daily* meet these eligibility criteria.

### Recruitment and randomization

A screening tool in our EMR used by our CPT consult service for older adults taking high-risk medications provides secure messaging to our research staff. Once eligibility is confirmed, the team ensures that a best possible medication history is completed to correct the home medication list side of our discharge medication reconciliation template. Once eligibility is cleared with the most responsible physician team (primary team), research staff will then approach the patient to introduce the study and complete the informed consent process which includes completing a short Capacity to Consent questionnaire (Appendix 2) [41]. Patients with cognitive impairment will not be excluded as they constitute a vulnerable group in need of assistance, but they must have a primary caregiver who assists them with medications and who provides informed consent if the patient is identified as not having capacity of consent [41]. For this pilot trial, either the patient or caregiver must be fluent in English.

Once patients have completed baseline assessments, they will be randomly allocated to the intervention or control arms in a 1:1 ratio in permuted blocks using a statistician-formulated randomization schedule produced in R and implemented in REDCap that will be available online to staff only at the time of randomization [42, 43]. To restrict treatment group imbalance, a maximal tolerable imbalance between treatment groups will be incorporated into the schedule [44].

### Intervention

**Fig. 2:**
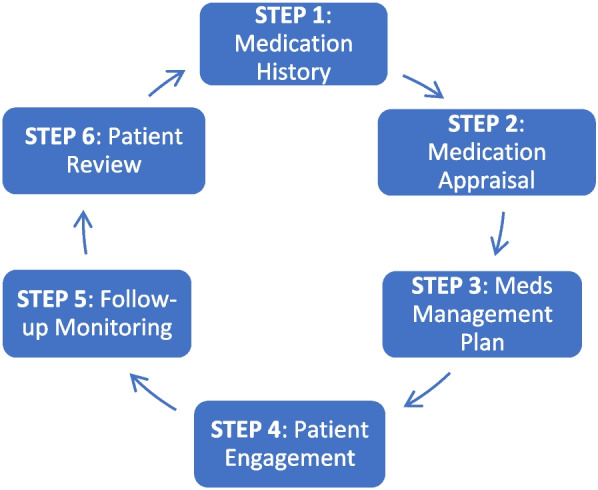
IMPROVE-IT HRM clinical approach

Our intervention follows the general innovative practices framework recommended by Health Quality Ontario for Transitions between Hospital and Home [45]. Fig. 2 shows a step-by-step clinical approach used for this intervention. Randomization to the intervention arm will trigger a request for a CPT (Clinical Pharmacology & Toxicology) consult. The initial CPT consult for each patient includes a comprehensive patient assessment including demographics, social situation, drug coverage insurance, functional status (activities of daily living, instrumental activities of daily living), cognition, frailty markers, level of caregiver involvement, past medical history and current problems, allergies and intolerances, detailed medication history including reminder aids and methods of accessing medication, physical exam, and review of current and historical laboratory and diagnostic imaging results. These details are mostly structured data items in the EMR which populates a customized CPT note template, which ensures some consistency of intervention across the participating consultants. We incorporate an admission medication reconciliation carried out by the ward pharmacist. The CPT consult will document a detailed “circle of care” for each patient (primary caregiver, hospital primary team, primary care team including family physician or nurse practitioner, community physician specialists and community pharmacists) and will identify all potential high-risk medications targets that the patient is taking or is due to resume post-hospitalization. Using patient preference elicitation methods and motivational interviewing, priorities for medication optimization will be negotiated [46–51]. Short patient infographics endorsed by the Canadian Medication Appropriateness and Deprescribing Network and largely addressing medication harms will be used as educational materials [52–61].

While the patient is still hospitalized, the high-risk medications care plan begins, and the team ensures close communication with the primary team, coordinates a detailed discharge medication reconciliation (documenting medication changes with rationale, formulating an accurate discharge prescription including rapid access to new medications), ensures circle-of-care communication, and sees the patient via virtual visit twice in follow-up at 1 week and 1 month after hospital discharge to complete and consolidate the care plan. Since this is a pragmatic trial, concomitant care is not prohibited. Pre-testing of the intervention with several patients has shown that the initial consult and two follow-up visits can be completed in less than 1 h each.

### Control

These patients will receive usual care by their primary team. This means that the primary team is responsible for coordinating medication management at discharge and posthospital follow-up, as is currently practiced.

### Outcomes

The Core Outcome Set for Interventions to Improve Polypharmacy in Older People was used to inform our selection of outcomes [62]. Core outcome sets are consensus-based guidelines from groups of clinicians, patients, and methodologists on which outcomes with which metrics are the most important to be measured in prospective studies [63]. In addition, we consulted other polypharmacy/deprescribing trials for recommended patient-important outcomes [64–69].

For this pilot RCT, we will analyze outcomes according to a set of primary and secondary outcomes (see details in Tables 1 and 2).

**Table 1:** IMPROVE-IT HRM outcomes [29, 30, 62, 64, 65, 70–73]

	**Outcome**
Feasibility	1. Participant recruitment rates
	2. Participant retention rate
	3. Trial resource utilization, costs
	4. Recommendation acceptance rates by primary team
	5. Recommendation acceptance rates by patient
	6. Recommendation adherence rates by patient
	7. CPT consultation volume capacity
	8. Potential to intervene entirely virtually (online)
Clinical	**1.** Medication management outcomes a) # inappropriate medications, # drug therapy problems improved, # high-risk medication problems improved, all measured by APEQ b) # medications per patient end study versus baseline
	**2.** Adverse drug events including adverse drug withdrawal event
	**3.** Medication errors, including preventability
	**4.** Patient problems with medications questionnaire (includes general adherence)
	**5.** Patient knowledge of medications
	**6.** Coordination and continuity of care
	**7.** Patient quality of life (EQ-5D-5L and medication-related QOL)
	**8.** Satisfaction with care (patients and providers)
	**9.** Health resource utilization, including clinical events of death, ED visits, hospitalizations, physician visits, other providers including diagnostics utilization, medication costs

**Table 2:** IMPROVE-IT HRM study outcomes and measures

	Outcomes	Criteria for success	Outcome measure	Method of analysis	When assessed
**A. Feasibility outcomes**					
A1. Primary feasibility outcomes	A1.1 Participant recruitment rates	≥ 30% of those eligible will be considered success	Percentage recruited and rate of recruitment (of those screened, of those eligible, and of those approached)	Counts (%) over time	Baseline
	A1.2 Participant retention rate	≥ 90% is considered success	Percentage of those recruited who complete at least end-study outcome	Counts (%) end study	End of study
	A1.3 Intervention adherence	≥ 90% is considered success	Percentage of those recruited who completed each phase of the study (baseline, virtual visits, end of study outcomes)	Counts (%) over time	End of study
	A1.4 Study resource utilization	< US $1500 per patient recruited and completing the study	Study costs per patient recruited to end follow-up	Cost of personnel, supplies, travel, etc. to run the study	End of study
A2. Secondary feasibility outcomes	A2.1 Clinical Pharmacology & Toxicology (CPT) Recommendation Acceptance — Primary Team	> 50% medication recommendations	% CPT recommendations per patient accepted (continued or implemented)	Counts (%) after consult	Hospital discharge
	A2.2 CPT recommendation acceptance — patients	> 50% medication recommendations	% CPT recommendations per patient that were initially followed by patient	Counts (%) after consult	Hospital discharge
	A2.3 CPT recommendation adherence — Patients	≥ 90% is considered success	% CPT recommendations per patient still being followed at end of study	Counts (%)	End of study
	A2.4 CPT consultation volume capacity	Number of eligible patients available to approach is > 3 per day	Volume of eligible patients available to approach per week	Counts	Baseline
	A2.5 Potential to intervene entirely virtually	> 90% patients can be managed through follow-up entirely by virtual care platform	Number (%) patients entering follow-up who do not require an unscheduled in-person visit Number of follow-up visits conducted using secure videoconference platform	Counts (%)	End of study
**B. Clinical outcomes**		**Hypothesis**			
B1. Primary clinical outcomes	B1.1 Drug therapy problems improved	Intervention group will have more improved drug therapy problems compared to control group (e.g., dose adjusted, discontinued, interacting drugs removed)	Percentage of baseline drug therapy problems identified by APEQ that have been improved by end of study	Mean difference in APEQ score changes	End of study
B2. Secondary clinical outcomes	B2.1 Adverse drug events	Intervention will have lower event rates	Event rate counts from end of study interviews using Leape and Bates scale	Counts (%)	End of study
	B2.2 Medication errors	Intervention will have fever medication errors	NCC-MERP definition	Counts (%)	End of study
	B2.3 Number of medications	Intervention will have more medications deprescribed per patient	Number of medications end study compared to baseline	Counts	End of study
	B2.4 Patient problems with medications	Intervention will have fewer medication problems, including better adherence	COMPETE Medication Problems Questionnaire	Difference in mean group scores	End of study
	B2.5 Medication knowledge - patient	Intervention will have higher scores	Medication knowledge assessment form	Difference in mean group scores	Baseline and Eed of study
	B2.6 Coordination and continuity of care	Intervention will have higher ratings	Coordination and Continuity of Care Questionnaire	Difference in mean group scores	End of study
	B2.7 Patient quality of life	Intervention will improve more	EQ5D-5L and MedQOL	Difference in mean group change scores	End of study
	B2.8 Patient satisfaction with care	Intervention will have higher scores	Patient Satisfaction Questionnaire	Descriptive analysis	End of study
	B2.9 Provider satisfaction with care	Intervention will have higher scores	Physician, pharmacist Study Satisfaction Questionnaire	Descriptive analysis	End of study
	B2.10 Cost-effectiveness/ health resource utilization	Intervention will be cost-effective using a threshold of US $50,000 per QALY	Cost per adverse drug events avoided and incremental cost per quality-adjusted life-years (QALYs) using EQ5D-5L utilities	Economic analysis	End of study

#### Primary outcomes

There are two types of primary outcomes that will be evaluated:

- The *primary feasibility outcomes* will be recruitment and retention rate for eligible patients and the estimated resources required per patient to complete the main trial (program delivery costs). We aim for at least 30% recruitment of those eligible, 90% retention of those recruited, and no more than US $1500 per patient spent on running the pilot trial. This figure is based on the limits of peer-reviewed RCT funding for a full trial.
- The *primary clinical outcome* will be the number of drug therapy problems remediated including the number of high-risk medications improved (for example, dose adjustment, discontinued, seriously interacting drugs removed) at 3-month posthospital discharge end-study visit, as judged by adjudicators using APEQ.

#### Secondary outcomes

- The *secondary feasibility outcomes* of CPT consultation include recommendation acceptance and adherence by primary team and by patients, consult volume capacity, and potential to apply the intervention entirely through virtual visits including eConsults. Thresholds for success are described in Table 2.
- *The secondary clinical/patient-important outcomes* will include the following:
  1. Adverse drug events (ADEs) defined as “Harm caused by exposure to a drug” adjudicated using Leape and Bates scale (6 - definitely due to medication, 5 - probably due to medication, 4 - possibly due to medication, 3 - possibly due to disease, 2 - probably due to disease, 1 - definitely due to disease, 5 and 6 considered ADE) [74]. ADE preventability will be evaluated using the recommended “best practice”-based definition [70].
  2. Medication errors defined as an error (of commission or omission) at any step along the pathway that begins with prescribing and ends with the patient taking the medication [74]. We will use the standard NCC-MERP classification system [71, 75].
  3. Number of medications per patient end study compared to baseline.
  4. Patient problems with medications: The COMPETE Medication Problems Questionnaire measures problems with medication access, handling, beliefs, and adherence [76].
  5. Medication knowledge assessment: This will be assessed using the Medication Knowledge Assessment form, which tests knowledge of medication name, indication, dosage instructions, and precautions [77].
  6. Coordination and continuity of care: Adapted from Health Quality Ontario’s draft guidance and a Rand instrument, the Coordination and Continuity of Care Questionnaire is designed to measure the quality of the transitional and follow-up care. We will focus on medication reconciliations and education and circle-of-care communications [78, 79].
  7. Patient quality of life: We will use the general EQ-5D-5L, a five-level measure of health status and utilities well validated in Canada and based on self-reported mobility, self-care, usual activities, pain discomfort, and anxiety/depression [80–82]. We will also use a “condition-specific” QOL measure, the medication-related quality-of-life measure which is designed for people living with polypharmacy [83].
  8. Satisfaction with care: Satisfaction reported by patients and by key health professionals is one of the recommended outcomes to report in medical research, as it may influence adherence [84, 85]. This outcome will be assessed by the Patient/Caregiver Study Satisfaction Survey and by the Provider Study Satisfaction Survey [86].
  9. Health resource utilization: This is a key outcome to determine cost-effectiveness and cost-utility which then determines whether healthcare systems might pay for this type of care [87, 88]. Using questionnaire and chart review, we will capture emergency department visits, hospitalizations, unplanned physician visits, and medication costs, including out-of-pocket medication costs in follow-up [79, 88].

Outcome data will be collected by research staff through a mix of patient interview and chart review. All adjudication will be carried out blinded to group allocation of the patient. Completeness of data for variables including sex, gender, age, social support, socioeconomic status, cognition, number of medications, and comorbidities will be examined as these are potential predictors of outcomes. Sensitivity analyses will assist with determination of potential for cost-effectiveness of the intervention overall and in selected subgroups.

Geographic wards in the hospital allow for evaluation of the intra-cluster correlation (ICC), which based on past experience, we expect to be low [76]. Additionally, barriers and facilitators to the success of the primary outcomes and potential for scalability to a larger trial will be assessed in weekly team meetings.

### Follow-up

Patients will be followed until 3-month posthospital discharge or until death or admission to long-term care home, whichever occurs first. Follow-up visits will be conducted by videoconference via Epic EMR or Ontario Telehealth Network, or by phone call, depending on the patient’s digital technology capability.

### Sample size

Since this is a pilot RCT, we will aim for 30 patients per group or 60 in total as this number is likely to provide adequate evidence regarding feasibility for ramp-up to a definitive trial.

### Blinding

As a pragmatic RCT layered on routine clinical care, it will not be possible to completely blind patients or their providers; however, outcome data collectors, adjudicators, and statisticians will be blinded to group allocation until analysis is completed at the end of the study.

### Data collection methods

Trained research staff will conduct interviews with the patients or caregivers, entering data electronically on study laptops directly into REDCap case report forms. The participants’ medical records will be reviewed to abstract data on baseline characteristics, medical history, and medication information. Strategies to promote participant retention and complete follow-up include reminding participants in advance of their end-of-study visit and communicating by email if email address is provided at baseline. Participants who drop out of the study will have their data to that point retained in the study, as approved by REB, to avoid bias. The reasons for study non-completion will be recorded. The SPIRIT figure outlining the schedule of enrolment, intervention, and assessment (as per evidence-based recommendations for the minimum content of a clinical trial protocol) can be found in Table 3 [89].

**Table 3:** IMPROVE-IT HRM schedule of enrolment, intervention, and assessment

**Assessment**	**Enrolment**	**Study in-progress**				**Study end**
	**Baseline**	**Inpatient stay**	**Hospital discharge**	**1 week**	**1 month**	**3 months**
Eligibility screen	A					
IMPROVE-IT assessment of capacity to consent	B					
Informed consent	1/C					
EQ-5D-5L v10- Canada	I/C					I/C
Medication quality of life (MedQOL)	1/C					I/C
Clinical Frailty Scale	I/C					I/C
Complete medication list (prescription, OTC, other)	I/C		I/C			I/C
Medication knowledge assessment	I/C					I/C
Circle-of-care information	I		I	I	I	
Randomization and allocation	I/C					
Clinical Pharmacology Toxicology Consult		I		I	I	
Appropriateness of prescribing (APEQ) — #drug therapy problems improved, #high-risk medication problems improved, #PIMs improved	I/C					I/C
Adverse drug event assessment						I/C
Patient problems with medications						I/C
Patient/caregiver satisfaction questionnaire						I/C
Provider satisfaction questionnaire						I/C
Health Resource Utilization Questionnaire						I/C

Abbreviations: *A*-all potentially eligible patients, *B-*all eligible and interested patients, *I*-intervention, *C*-control, *I/C*-both groups

### Data management, privacy, and confidentiality

REDCap’s secure, web-based platform used widely internationally, providing interfaces for validated data capture, role-specific access, audit trails for tracking data manipulation and exports, automated export procedures to SAS, and encrypted transmissions [90, 91]. Paper study documents such as signed informed consent forms will be stored in our secure research office once they are scanned into REDCap study files. Regular data quality checks, such as automatic range checks, will be performed by the study team to identify data that appear inconsistent, incomplete, or inaccurate.

Patients will not be identifiable in the project results database. The identifying information required for the clinical team to deliver the intervention will be kept in a separate database. Access to the final dataset will be restricted to the core research team.

### Statistical analyses

The reporting of the results of this trial will follow the CONSORT extension for pilot trials [92]. We will use descriptive statistics for presentation of baseline variables and adequacy of follow-up. Feasibility analysis, including recruitment rate (at least 30% of those eligible is considered success), participant retention rate (≥ 90% to end of study is considered success), and study resource utilization required (less than US $1500 CAD per patient recruited), will be descriptive.

Analysis will use intention-to-treat methods with censoring only if the patient dies or drops out of the study with refusal of negotiated further assessments. Costs and quality-adjusted life-years (QALYs) associated with the two interventions will be determined from a payer and societal perspective. Healthcare resource utilization collected as part of the trial will be costed using public data sources. Using information from the EQ-5D-5L, quality-adjusted life-years (QALYs) will be determined under an area under the curve approach.

Research staff and statisticians will review outcome data and analysis blinded to group identification. Given the short follow-up, low risk of the trial, and pilot design, no interim analysis or imputation for missing data is planned. All statistical analyses will be performed using SAS V9.4 software (SAS Institute Inc., Cary, NC, USA).

### Data monitoring

Any serious adverse event will be reviewed by our Trial Steering Committee (TSC) within 48 h of detection, to discern any attribution to our procedures. If found to be due to our coordination procedures, the Trial Steering Committee will recommend whether modifications are indicated. The TSC will be composed of individuals with expertise in clinical trials, chaired by the lead statistician, and include the PI, the operational statistician, plus a methodologist independent of the study team. Similarly, since this is a short pilot pragmatic RCT where no harm is expected and adjustment of trial procedures may be necessary for feasibility, no formal external auditing of trial conduct is planned. There is no requirement for additional ancillary and posttrial care for those who might come to harm while in the trial, as usual medical care which covers this eventuality is already in place.

#### Impact of research

By creating a network of clinicians, patients, researchers, drug policy advisors, information technology advisors, quality improvement advisors, and hospital and national medication safety administration, we hope to strengthen and broaden the usual dissemination of research to practice We expect the pilot study to inform a future definitive trial by identifying solutions to overcome potential limitations including the following:

- The CPT team is a scarce resource which limits eventual generalizability. Based on pilot results, we will plan to study how physician assistants and clinical pharmacists can be trained to do this work.
- The short follow-up of 3 months is typical of transitional care initiatives but may be too short to show impact on clinical outcomes.
- The single-center design aids feasibility of the pilot but limits generalizability. A larger trial, if feasible, will aim to recruit other hospitals using the Epic EMR or members of the national deprescribing network.
- A definitive trial will highlight clinical outcomes as primary outcomes.

### Supplementary Information

**Additional file 1: Appendix 1.** IMPROVE-IT HRM high-risk medications [27, 28].

**Additional file 2: Appendix 2.** IMPROVE-IT HRM Capacity to Consent Questionnaire.

## Data Availability

An anonymized dataset will be shared in accordance with future requirements of our funders, the Canadian Institutes of Health Research.

## Abbreviations

ADE: Adverse drug event
APEQ: Appropriateness of Prescribing Evaluation Questionnaire
CPT: Clinical pharmacology and toxicology
EMR: Electronic medical records
EQ-5D-5L: European Quality-of-Life Five Dimension
HRM: High-risk medication
NCC-MERP: National Coordinating Council for Medication Error Reporting and Prevention
PIM: Potentially inappropriate medication
QOL: Quality of life
RCT: Randomized control trial
REDCap: Research Electronic Data Capture
SD: Standard deviation
STOPP: Screening Tool of Older People’s Prescriptions
WHO: World Health Organization
