# Supplementary material for: Improving medication prescribing-related outcomes for vulnerable elderly in transitions on high-risk medications (IMPROVE-IT HRM): a pilot randomized trial protocol
Source: Pilot Feasibility Stud. 2024 Apr 10;10:60. doi: 10.1186/s40814-024-01484-6 (PMC11005201; doi:10.1186/s40814-024-01484-6)
Supplement: Supplementary file 1 — Additional file 1: Appendix 1. IMPROVE-IT HRM high-risk medications [27, 28]. [file 40814_2024_1484_MOESM1_ESM.docx]

## Appendix 1 IMPROVE-IT HRM High-Risk Medications^27, 28^

| **Medication Classes** | **Medications** | **ATC Codes** |
| --- | --- | --- |
| 1. Glucose lowering/hypoglycemics 2. GI Medications | Insulin human | A10AB01 |
|  | Insulin pork | A10AB03 |
|  | Insulin lispro | A10AB04 |
|  | Insulin aspart | A10AB05 |
|  | Insulin glulisine | A10AB06 |
|  | Insulin human (intermediate acting) | A10AC01 |
|  | Insulin pork (intermediate acting) | A10AC03 |
|  | Insulin human (long acting) | A10AD01 |
|  | Insulin lispro (long acting) | A10AD04 |
|  | Insulin aspart (long acting) | A10AD05 |
|  | Glibenclamide | A10BB01 |
|  | Chlorpropamide | A10BB02 |
|  | Tolbutamide | A10BB03 |
|  | Gliclazide | A10BB09 |
|  | Glimepiride | A10BB12 |
|  | Acarbose | A10BF01 |
|  | Rosiglitazone | A10BG02 |
|  | Pioglitazone | A10BG03 |
|  | Sitagliptin | A10BH01 |
|  | Saxagliptin | A10BH03 |
|  | Alogliptin | A10BH04 |
|  | Linagliptin | A10BH05 |
|  | Liraglutide | A10BJ02 |
|  | Lixisenatide | A10BJ03 |
|  | Dulaglutide | A10BJ05 |
|  | Semaglutide | A10BJ06 |
|  | Dapagliflozin | A10BK01 |
|  | Canagliflozin | A10BK02 |
|  | Empagliflozin | A10BK03 |
|  | Repaglinide | A10BX02 |
|  | Tirzepatide | A10BX16 |
|  | Papaverine | A03AD01 |
|  | Metoclopramide | A03FA01 |
|  | Domperidone | A03FA03 |
| 1. Anti-thrombotics | Warfarin | B01AA03 |
|  | Acenocoumarol | B01AA07 |
|  | Heparin | B01AB01 |
|  | Enoxaparin | B01AB05 |
|  | Nadroparin | B01AB06 |
|  | Tinzaparin | B01AB10 |
|  | Argatroban | B01AE03 |
|  | Bivalirudin | B01AE06 |
|  | Dabigatran etexilate | B01AE07 |
|  | Rivaroxaban | B01AF01 |
|  | Apixaban | B01AF02 |
|  | Edoxaban | B01AF03 |
|  | Fondaparinux | B01AX05 |
| 1. Cardiac Medications | | |
| - 1. Digoxin | Digoxin | C01AA05 |
| - 1. Amiodarone | Amiodarone | C01BD01 |
| - 1. Diuretics | Hydrochlorothiazide | C03AA03 |
|  | Chlorthalidone | C03BA04 |
|  | Metolazone | C03BA08 |
|  | Indapamide | C03BA11 |
|  | Furosemide | C03CA01 |
|  | Bumetanide | C03CA02 |
|  | Etacrynic acid | C03CC01 |
|  | Spironolactone | C03DA01 |
|  | Eplerenone | C03DA04 |
|  | Amiloride | C03DB01 |
|  | Tolvaptan | C03XA01 |
| 1. Hormone therapy | | |
| - 1. Androgens for menopause | Methyltestosterone | G03BA02 |
|  | Testosterone | G03BA03 |
| - 1. Postmenopausal estrogen | Estradiol | G03CA03 |
|  | Estrone | G03CA07 |
|  | Dienestrol | G03CB01 |
|  | Tibolone | G03CX01 |
| 1. Oxybutinin | Oxybutynin | G04BD04 |
| 1. Analgesics | | |
| - 1. Glucocorticoids | Betamethasone | H02AB01 |
|  | Dexamethasone | H02AB02 |
|  | Methylprednisolone | H02AB04 |
|  | Prednisolone | H02AB06 |
|  | Prednisone | H02AB07 |
|  | Triamcinolone | H02AB08 |
|  | Hydrocortisone | H02AB09 |
|  | Cortisone | H02AB10 |
| - 1. Colchicine (long term use) | Colchicine | M04AC01 |
| - 1. Opioids | Morphine | N02AA01 |
|  | Hydromorphone | N02AA03 |
|  | Oxycodone | N02AA05 |
|  | Oxycodone and naloxone | N02AA55 |
|  | Codeine, combinations excl. psycholeptics | N02AA59 |
|  | Codeine, combinations with psycholeptics | N02AA79 |
|  | Fentanyl | N02AB03 |
|  | Buprenorphine | N02AE01 |
|  | Butorphanol | N02AF01 |
|  | Nalbuphine | N02AF02 |
|  | Codeine and paracetamol | N02AJ06 |
|  | Tramadol and paracetamol | N02AJ13 |
|  | Oxycodone and paracetamol | N02AJ17 |
|  | Oxycodone and acetylsalicylic acid | N02AJ18 |
|  | Tramadol | N02AX02 |
|  | Tapentadol | N02AX06 |
|  | Methadone | N07BC02 |
|  | Diamorphine | N07BC06 |
|  | Hydrocodone | R05DA03 |
|  | Codeine | R05DA04 |
| 1. Antimicrobials   (only if intended for long-term use post-discharge – more than 4 weeks total therapy) | Doxycycline | J01AA02 |
|  | Tetracycline | J01AA07 |
|  | Minocycline | J01AA08 |
|  | Tigecycline | J01AA12 |
|  | Chloramphenicol | J01BA01 |
|  | Ampicillin | J01CA01 |
|  | Amoxicillin | J01CA04 |
|  | Piperacillin | J01CA12 |
|  | Benzylpenicillin | J01CE01 |
|  | Phenoxymethylpenicillin | J01CE02 |
|  | Procaine benzylpenicillin | J01CE09 |
|  | Cloxacillin | J01CF02 |
|  | Oxacillin | J01CF04 |
|  | Amoxicillin and beta-lactamase inhibitor | J01CR02 |
|  | Piperacillin and beta-lactamase inhibitor | J01CR05 |
|  | Cefalexin | J01DB01 |
|  | Cefalotin | J01DB03 |
|  | Cefazolin | J01DB04 |
|  | Cefadroxil | J01DB05 |
|  | Cefotaxime | J01DD01 |
|  | Ceftazidime | J01DD02 |
|  | Ceftriaxone | J01DD04 |
|  | Cefixime | J01DD08 |
|  | Cefepime | J01DE01 |
|  | Aztreonam | J01DF01 |
|  | Meropenem | J01DH02 |
|  | Ertapenem | J01DH03 |
|  | Ceftobiprole medocaril | J01DI01 |
|  | Ceftolozane and beta-lactamase inhibitor | J01DI54 |
|  | Imipenem and cilastatin | J01DH51 |
|  | Sulfamethizole | J01EB02 |
|  | Sulfapyridine | J01EB04 |
|  | Sulfadiazine | J01EC02 |
|  | Sulfamethoxazole and trimethoprim | J01EE01 |
|  | Erythromycin | J01FA01 |
|  | Spiramycin | J01FA02 |
|  | Clarithromycin | J01FA09 |
|  | Azithromycin | J01FA10 |
|  | Clindamycin | J01FF01 |
|  | Streptomycin | J01GA01 |
|  | Tobramycin | J01GB01 |
|  | Gentamicin | J01GB03 |
|  | Amikacin | J01GB06 |
|  | Ciprofloxacin | J01MA02 |
|  | Norfloxacin | J01MA06 |
|  | Levofloxacin | J01MA12 |
|  | Moxifloxacin | J01MA14 |
|  | Vancomycin | J01XA01 |
|  | Telavancin | J01XA03 |
|  | Dalbavancin | J01XA04 |
|  | Colistin | J01XB01 |
|  | Polymyxin B | J01XB02 |
|  | Metronidazole | J01XD01 |
|  | Nitrofurantoin | J01XE01 |
|  | Fosfomycin | J01XX01 |
|  | Spectinomycin | J01XX04 |
|  | Methenamine | J01XX05 |
|  | Linezolid | J01XX08 |
|  | Daptomycin | J01XX09 |
|  | Bacitracin | J01XX10 |
|  | Tedizolid | J01XX11 |
|  | Lefamulin | J01XX12 |
|  | Amphotericin B | J02AA01 |
|  | Ketoconazole | J02AB02 |
|  | Fluconazole | J02AC01 |
|  | Itraconazole | J02AC02 |
|  | Voriconazole | J02AC03 |
|  | Posaconazole | J02AC04 |
|  | Isavuconazole | J02AC05 |
|  | Caspofungin | J02AX04 |
|  | Micafungin | J02AX05 |
|  | Anidulafungin | J02AX06 |
|  | Rifampicin | J04AB02 |
|  | Rifabutin | J04AB04 |
|  | Isoniazid | J04AC01 |
|  | Pyrazinamide | J04AK01 |
|  | Ethambutol | J04AK02 |
|  | Dapsone | J04BA02 |
|  | Acyclovir | J05AB01 |
|  | Ganciclovir | J05AB06 |
|  | Famciclovir | J05AB09 |
|  | Valaciclovir | J05AB11 |
|  | Cidofovir | J05AB12 |
|  | Valganciclovir | J05AB14 |
|  | Remdesivir | J05AB16 |
|  | Foscarnet | J05AD01 |
|  | Ritonavir | J05AE03 |
|  | Fosamprenavir | J05AE07 |
|  | Atazanavir | J05AE08 |
|  | Tipranavir | J05AE09 |
|  | Darunavir | J05AE10 |
|  | Nirmatrelvir and ritonavir | J05AE30 |
|  | Zidovudine | J05AF01 |
|  | Lamivudine | J05AF05 |
|  | Abacavir | J05AF06 |
|  | Tenofovir disoproxil | J05AF07 |
|  | Adefovir dipivoxil | J05AF08 |
|  | Entecavir | J05AF10 |
|  | Tenofovir alafenamide | J05AF13 |
|  | Nevirapine | J05AG01 |
|  | Efavirenz | J05AG03 |
|  | Etravirine | J05AG04 |
|  | Rilpivirine | J05AG05 |
|  | Doravirine | J05AG06 |
|  | Zanamivir | J05AH01 |
|  | Oseltamivir | J05AH02 |
|  | Raltegravir | J05AJ01 |
|  | Dolutegravir | J05AJ03 |
|  | Cabotegravir | J05AJ04 |
|  | Ribavirin | J05AP01 |
|  | Sofosbuvir | J05AP08 |
|  | Sofosbuvir and ledipasvir | J05AP51 |
|  | Sofosbuvir and velpatasvir | J05AP55 |
|  | Sofosbuvir, velpatasvir and voxilaprevir | J05AP56 |
|  | Glecaprevir and pibrentasvir | J05AP57 |
|  | Zidovudine and lamivudine | J05AR01 |
|  | Lamivudine and abacavir | J05AR02 |
|  | Tenofovir disoproxil and emtricitabine | J05AR03 |
|  | Zidovudine, lamivudine and nevirapine | J05AR05 |
|  | Emtricitabine, tenofovir disoproxil and efavirenz | J05AR06 |
|  | Emtricitabine, tenofovir disoproxil and rilpivirine | J05AR08 |
|  | Emtricitabine, tenofovir disoproxil, elvitegravir and cobicistat | J05AR09 |
|  | Lopinavir and ritonavir | J05AR10 |
|  | Lamivudine, abacavir and dolutegravir | J05AR13 |
|  | Darunavir and cobicistat | J05AR14 |
|  | Emtricitabine and tenofovir alafenamide | J05AR17 |
|  | Emtricitabine, tenofovir alafenamide, elvitegravir and cobicistat | J05AR18 |
|  | Emtricitabine, tenofovir alafenamide and rilpivirine | J05AR19 |
|  | Emtricitabine, tenofovir alafenamide and bictegravir | J05AR20 |
|  | Dolutegravir and rilpivirine | J05AR21 |
|  | Emtricitabine, tenofovir alafenamide, darunavir and cobicistat | J05AR22 |
|  | Lamivudine, tenofovir disoproxil and doravirine | J05AR24 |
|  | Lamivudine and dolutegravir | J05AR25 |
|  | Inosine pranobex | J05AX05 |
|  | Enfuvirtide | J05AX07 |
|  | Maraviroc | J05AX09 |
|  | Maribavir | J05AX10 |
|  | Letermovir | J05AX18 |
|  | Tecovirimat | J05AX24 |
|  | Baloxavir marboxil | J05AX25 |
|  | Fostemsavir | J05AX29 |
|  | Lenacapavir | J05AX31 |
| 1. Antineoplastics | Cyclophosphamide | L01AA01 |
|  | Chlorambucil | L01AA02 |
|  | Melphalan | L01AA03 |
|  | Chlormethine | L01AA05 |
|  | Ifosfamide | L01AA06 |
|  | Bendamustine | L01AA09 |
|  | Busulfan | L01AB01 |
|  | Treosulfan | L01AB02 |
|  | Thiotepa | L01AC01 |
|  | Carmustine | L01AD01 |
|  | Lomustine | L01AD02 |
|  | Temozolomide | L01AX03 |
|  | Dacarbazine | L01AX04 |
|  | Methotrexate | L01BA01 |
|  | Raltitrexed | L01BA03 |
|  | Pemetrexed | L01BA04 |
|  | Pralatrexate | L01BA05 |
|  | Mercaptopurine | L01BB02 |
|  | Tioguanine | L01BB03 |
|  | Cladribine | L01BB04 |
|  | Fludarabine | L01BB05 |
|  | Clofarabine | L01BB06 |
|  | Nelarabine | L01BB07 |
|  | Cytarabine | L01BC01 |
|  | Fluorouracil | L01BC02 |
|  | Gemcitabine | L01BC05 |
|  | Capecitabine | L01BC06 |
|  | Azacitidine | L01BC07 |
|  | Decitabine | L01BC08 |
|  | Vinblastine | L01CA01 |
|  | Vincristine | L01CA02 |
|  | Vindesine | L01CA03 |
|  | Vinorelbine | L01CA04 |
|  | Paclitaxel | L01CD01 |
|  | Docetaxel | L01CD02 |
|  | Cabazitaxel | L01CD04 |
|  | Topotecan | L01CE01 |
|  | Irinotecan | L01CE02 |
|  | Trabectedin | L01CX01 |
|  | Dactinomycin | L01DA01 |
|  | Doxorubicin | L01DB01 |
|  | Daunorubicin | L01DB02 |
|  | Epirubicin | L01DB03 |
|  | Idarubicin | L01DB06 |
|  | Mitoxantrone | L01DB07 |
|  | Bleomycin | L01DC01 |
|  | Mitomycin | L01DC03 |
|  | Imatinib | L01EA01 |
|  | Dasatinib | L01EA02 |
|  | Nilotinib | L01EA03 |
|  | Bosutinib | L01EA04 |
|  | Ponatinib | L01EA05 |
|  | Asciminib | L01EA06 |
|  | Gefitinib | L01EB01 |
|  | Erlotinib | L01EB02 |
|  | Afatinib | L01EB03 |
|  | Osimertinib | L01EB04 |
|  | Dacomitinib | L01EB07 |
|  | Vemurafenib | L01EC01 |
|  | Dabrafenib | L01EC02 |
|  | Encorafenib | L01EC03 |
|  | Crizotinib | L01ED01 |
|  | Ceritinib | L01ED02 |
|  | Alectinib | L01ED03 |
|  | Brigatinib | L01ED04 |
|  | Lorlatinib | L01ED05 |
|  | Trametinib | L01EE01 |
|  | Cobimetinib | L01EE02 |
|  | Binimetinib | L01EE03 |
|  | Selumetinib | L01EE04 |
|  | Palbociclib | L01EF01 |
|  | Ribociclib | L01EF02 |
|  | Abemaciclib | L01EF03 |
|  | Temsirolimus | L01EG01 |
|  | Everolimus | L01EG02 |
|  | Lapatinib | L01EH01 |
|  | Neratinib | L01EH02 |
|  | Tucatinib | L01EH03 |
|  | Ruxolitinib | L01EJ01 |
|  | Fedratinib | L01EJ02 |
|  | Axitinib | L01EK01 |
|  | Ibrutinib | L01EL01 |
|  | Acalabrutinib | L01EL02 |
|  | Zanubrutinib | L01EL03 |
|  | Idelalisib | L01EM01 |
|  | Alpelisib | L01EM03 |
|  | Erdafitinib | L01EN01 |
|  | Pemigatinib | L01EN02 |
|  | Infigratinib | L01EN03 |
|  | Sunitinib | L01EX01 |
|  | Sorafenib | L01EX02 |
|  | Pazopanib | L01EX03 |
|  | Vandetanib | L01EX04 |
|  | Regorafenib | L01EX05 |
|  | Cabozantinib | L01EX07 |
|  | Lenvatinib | L01EX08 |
|  | Nintedanib | L01EX09 |
|  | Midostaurin | L01EX10 |
|  | Larotrectinib | L01EX12 |
|  | Gilteritinib | L01EX13 |
|  | Entrectinib | L01EX14 |
|  | Capmatinib | L01EX17 |
|  | Ripretinib | L01EX19 |
|  | Tepotinib | L01EX21 |
|  | Selpercatinib | L01EX22 |
|  | Pralsetinib | L01EX23 |
|  | Rituximab | L01FA01 |
|  | Ofatumumab | L01FA02 |
|  | Obinutuzumab | L01FA03 |
|  | Inotuzumab ozogamicin | L01FB01 |
|  | Daratumumab | L01FC01 |
|  | Isatuximab | L01FC02 |
|  | Trastuzumab | L01FD01 |
|  | Pertuzumab | L01FD02 |
|  | Trastuzumab emtansine | L01FD03 |
|  | Trastuzumab deruxtecan | L01FD04 |
|  | Cetuximab | L01FE01 |
|  | Panitumumab | L01FE02 |
|  | Necitumumab | L01FE03 |
|  | Nivolumab | L01FF01 |
|  | Pembrolizumab | L01FF02 |
|  | Durvalumab | L01FF03 |
|  | Avelumab | L01FF04 |
|  | Atezolizumab | L01FF05 |
|  | Cemiplimab | L01FF06 |
|  | Dostarlimab | L01FF07 |
|  | Bevacizumab | L01FG01 |
|  | Ramucirumab | L01FG02 |
|  | Gemtuzumab ozogamicin | L01FX02 |
|  | Ipilimumab | L01FX04 |
|  | Brentuximab vedotin | L01FX05 |
|  | Dinutuximab | L01FX06 |
|  | Blinatumomab | L01FX07 |
|  | Elotuzumab | L01FX08 |
|  | Mogamulizumab | L01FX09 |
|  | Tafasitamab | L01FX12 |
|  | Enfortumab vedotin | L01FX13 |
|  | Polatuzumab vedotin | L01FX14 |
|  | Sacituzumab govitecan | L01FX17 |
|  | Amivantamab | L01FX18 |
|  | Cisplatin | L01XA01 |
|  | Carboplatin | L01XA02 |
|  | Oxaliplatin | L01XA03 |
|  | Procarbazine | L01XB01 |
|  | Porfimer sodium | L01XD01 |
|  | Methyl aminolevulinate | L01XD03 |
|  | Aminolevulinic acid | L01XD04 |
|  | Tretinoin | L01XF01 |
|  | Bortezomib | L01XG01 |
|  | Carfilzomib | L01XG02 |
|  | Ixazomib | L01XG03 |
|  | Vorinostat | L01XH01 |
|  | Romidepsin | L01XH02 |
|  | Vismodegib | L01XJ01 |
|  | Sonidegib | L01XJ02 |
|  | Glasdegib | L01XJ03 |
|  | Olaparib | L01XK01 |
|  | Niraparib | L01XK02 |
|  | Talazoparib | L01XK04 |
|  | Axicabtagene ciloleucel | L01XL03 |
|  | Tisagenlecleucel | L01XL04 |
|  | Brexucabtagene autoleucel | L01XL06 |
|  | Idecabtagene vicleucel | L01XL07 |
|  | Amsacrine | L01XX01 |
|  | Asparaginase | L01XX02 |
|  | Altretamine | L01XX03 |
|  | Hydroxycarbamide | L01XX05 |
|  | Pentostatin | L01XX08 |
|  | Estramustine | L01XX11 |
|  | Mitotane | L01XX23 |
|  | Pegaspargase | L01XX24 |
|  | Arsenic trioxide | L01XX27 |
|  | Anagrelide | L01XX35 |
|  | Eribulin | L01XX41 |
|  | Aflibercept | L01XX44 |
|  | Venetoclax | L01XX52 |
|  | Enasidenib | L01XX59 |
|  | Selinexor | L01XX66 |
|  | Lurbinectedin | L01XX69 |
|  | Sotorasib | L01XX73 |
|  | Belzutifan | L01XX74 |
|  | Venetoclax | L01XX52 |
|  | Tebentafusp | L01XX75 |
|  | Buserelin | L02AE01 |
|  | Leuprorelin | L02AE02 |
|  | Goserelin | L02AE03 |
|  | Triptorelin | L02AE04 |
|  | Tamoxifen | L02BA01 |
|  | Fulvestrant | L02BA03 |
|  | Nilutamide | L02BB02 |
|  | Bicalutamide | L02BB03 |
|  | Enzalutamide | L02BB04 |
|  | Apalutamide | L02BB05 |
|  | Darolutamide | L02BB06 |
| 1. Immunosuppressants | Antilymphocyte immunoglobulin (horse) | L04AA03 |
|  | Antithymocyte immunoglobulin (rabbit) | L04AA04 |
|  | Mycophenolic acid | L04AA06 |
|  | Sirolimus | L04AA10 |
|  | Leflunomide | L04AA13 |
|  | Everolimus | L04AA18 |
|  | Natalizumab | L04AA23 |
|  | Abatacept | L04AA24 |
|  | Eculizumab | L04AA25 |
|  | Belimumab | L04AA26 |
|  | Fingolimod | L04AA27 |
|  | Tofacitinib | L04AA29 |
|  | Teriflunomide | L04AA31 |
|  | Apremilast | L04AA32 |
|  | Vedolizumab | L04AA33 |
|  | Alemtuzumab | L04AA34 |
|  | Ocrelizumab | L04AA36 |
|  | Baricitinib | L04AA37 |
|  | Ozanimod | L04AA38 |
|  | Cladribine | L04AA40 |
|  | Siponimod | L04AA42 |
|  | Ravulizumab | L04AA43 |
|  | Upadacitinib | L04AA44 |
|  | Belumosudil | L04AA48 |
|  | Ponesimod | L04AA50 |
|  | Anifrolumab | L04AA51 |
|  | Ofatumumab | L04AA52 |
|  | Pegcetacoplan | L04AA54 |
|  | Deucravacitinib | L04AA56 |
|  | Avacopan | L04AA59 |
|  | Etanercept | L04AB01 |
|  | Infliximab | L04AB02 |
|  | Adalimumab | L04AB04 |
|  | Certolizumab pegol | L04AB05 |
|  | Golimumab | L04AB06 |
|  | Basiliximab | L04AC02 |
|  | Anakinra | L04AC03 |
|  | Ustekinumab | L04AC05 |
|  | Tocilizumab | L04AC07 |
|  | Canakinumab | L04AC08 |
|  | Secukinumab | L04AC10 |
|  | Siltuximab | L04AC11 |
|  | Brodalumab | L04AC12 |
|  | Ixekizumab | L04AC13 |
|  | Sarilumab | L04AC14 |
|  | Guselkumab | L04AC16 |
|  | Tildrakizumab | L04AC17 |
|  | Risankizumab | L04AC18 |
|  | Satralizumab | L04AC19 |
|  | Bimekizumab | L04AC21 |
|  | Spesolimab | L04AC22 |
|  | Ciclosporin | L04AD01 |
|  | Tacrolimus | L04AD02 |
|  | Azathioprine | L04AX01 |
|  | Thalidomide | L04AX02 |
|  | Methotrexate | L04AX03 |
|  | Lenalidomide | L04AX04 |
|  | Pirfenidone | L04AX05 |
|  | Pomalidomide | L04AX06 |
|  | Dimethyl fumarate | L04AX07 |
| 1. Muscle relaxant | Baclofen | M03BX01 |
|  | Methocarbamol | M03BA03 |
|  | Methocarbamol, combinations excl. psycholeptics | M03BA53 |
|  | Chlorzoxazone, combinations excl. psycholeptics | M03BB53 |
|  | Orphenadrine (citrate) | M03BC01 |
|  | Tizanidine | M03BX02 |
|  | Cyclobenzaprine | M03BX08 |
|  | Dantrolene | M03CA01 |
| 1. Antipsychotics | Chlorpromazine | N05AA01 |
|  | Methotrimeprazine | N05AA02 |
|  | Fluphenazine | N05AB02 |
|  | Perphenazine | N05AB03 |
|  | Prochlorperazine | N05AB04 |
|  | Trifluoperazine | N05AB06 |
|  | Periciazine | N05AC01 |
|  | Haloperidol | N05AD01 |
|  | Ziprasidone | N05AE04 |
|  | Lurasidone | N05AE05 |
|  | Flupentixol | N05AF01 |
|  | Zuclopenthixol | N05AF05 |
|  | Pimozide | N05AG02 |
|  | Loxapine | N05AH01 |
|  | Clozapine | N05AH02 |
|  | Olanzapine | N05AH03 |
|  | Quetiapine | N05AH04 |
|  | Asenapine | N05AH05 |
|  | Risperidone | N05AX08 |
|  | Aripiprazole | N05AX12 |
|  | Paliperidone | N05AX13 |
|  | Cariprazine | N05AX15 |
|  | Brexpiprazole | N05AX16 |
| 13. Sedatives and hypnotics | | |
| 13.1. BZRAs | Clonazepam | N03AE01 |
|  | Diazepam | N05BA01 |
|  | Lorazepam | N05BA06 |
|  | Alprazolam | N05BA12 |
|  | Flurazepam | N05CD01 |
|  | Nitrazepam | N05CD02 |
|  | Triazolam | N05CD05 |
|  | Temazepam | N05CD07 |
|  | Midazolam | N05CD08 |
|  | Zopiclone | N05CF01 |
|  | Zolpidem | N05CF02 |
|  | Eszopiclone | N05CF04 |
| 13.3. Sedating Antihistamines | Hydroxyzine | N05BB01 |
|  | Diphenhydramine | R06AA02 |
|  | Dimenhydrinate | R06AA11 |
|  | Chlorpheniramine | R06AB04 |
|  | Alimemazine | R06AD01 |
|  | Promethazine | R06AD02 |
|  | Cetirizine | R06AE07 |
|  | Cyproheptadine | R06AX02 |
|  | Ketotifen | R06AX17 |
| 1. Lithium | Lithium | N05AN01 |
| 1. Tricyclic antidepressants | Desipramine | N06AA01 |
|  | Imipramine | N06AA02 |
|  | Clomipramine | N06AA04 |
|  | Trimipramine | N06AA06 |
|  | Amitriptyline | N06AA09 |
|  | Nortriptyline | N06AA10 |
|  | Doxepin | N06AA12 |
| 1. Trazodone | Trazodone | N06AX05 |
| 1. Acetylcholinesterase inhibitors | Donepezil | N06DA02 |
|  | Rivastigmine | N06DA03 |
|  | Galantamine | N06DA04 |
|  | Neostigmine | N07AA01 |
|  | Pyridostigmine | N07AA02 |
| 1. Other (quinine, supplements melatonin, etc) | Melatonin | N05CH01 |
|  | Quinine | P01BC01 |
| 1. Aminoquinolines | Hydroxychloroquine | P01BA02 |
|  | Primaquine | P01BA03 |
