# Supplementary figures and images for: Improving medication prescribing-related outcomes for vulnerable elderly in transitions on high-risk medications (IMPROVE-IT HRM): a pilot randomized trial protocol
Source: Pilot Feasibility Stud. 2024 Apr 10;10:60. doi: 10.1186/s40814-024-01484-6 (PMC11005201; doi:10.1186/s40814-024-01484-6)

##
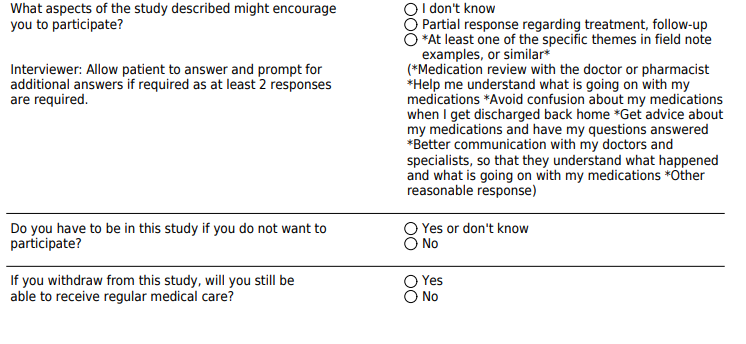

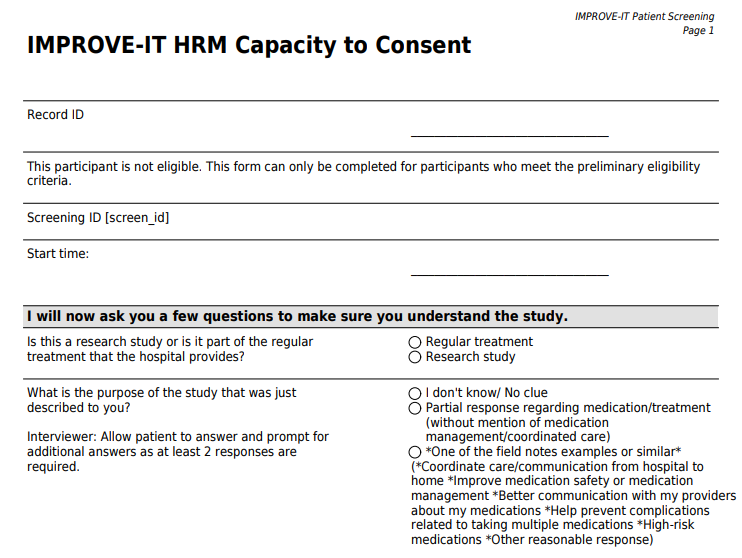
Appendix 2. IMPROVE-IT HRM Capacity to Consent Questionnaire


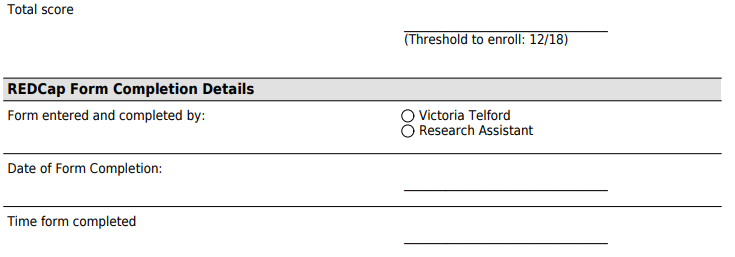

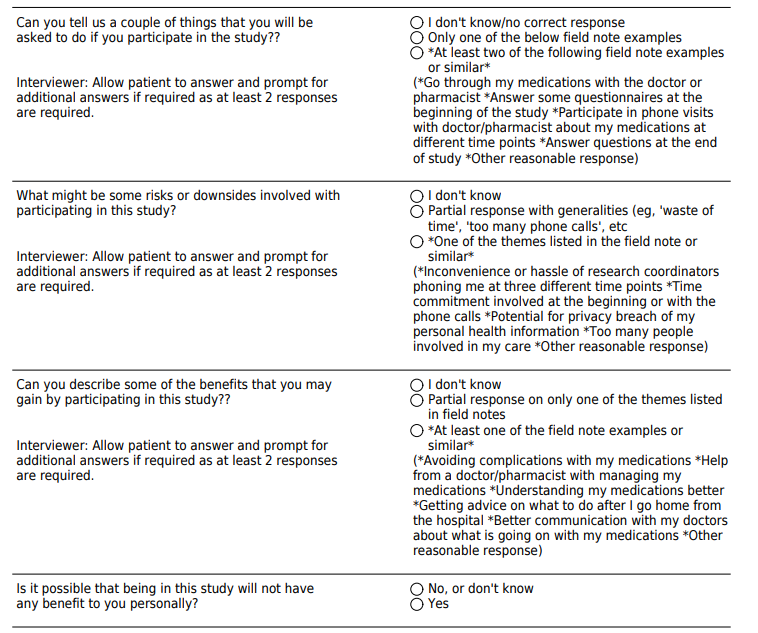

Supplement: Supplementary file 2 — Additional file 2: Appendix 2. IMPROVE-IT HRM Capacity to Consent Questionnaire. [file 40814_2024_1484_MOESM2_ESM.docx]
